# Collimated flat-top beam shaper metasurface doublet based on the complex-amplitude constraint Gerchberg–Saxton algorithm

**DOI:** 10.1515/nanoph-2023-0719

**Published:** 2024-01-11

**Authors:** Dongbai Xue, Xiong Dun, Zeyong Wei, DongDong Li, Jingyuan Zhu, Zhanyi Zhang, Zhanshan Wang, Xinbin Cheng

**Affiliations:** Institute of Precision Optical Engineering, School of Physics Science and Engineering, Tongji University, Shanghai 200092, China; MOE Key Laboratory of Advanced Micro-Structured Materials, Shanghai 200092, China; Shanghai Frontiers Science Center of Digital Optics, Shanghai 200092, China; Shanghai Professional Technical Service Platform for Full-Spectrum and High-Performance Optical Thin Film Devices and Applications, Shanghai 200092, China; Shanghai Institute of Intelligent Science and Technology, Tongji University, Shanghai 200092, China

**Keywords:** beam shaping, collimated flat-top beam, metasurface doublet, complex-amplitude constraint, Gerchberg–Saxton algorithm

## Abstract

Collimated flat-top beam shapers primarily consisting of freeform lenses have a wide range of applications and pose challenges in terms of processing and integration when the diameter is less than millimeters. Metasurfaces represent a promising solution to planarize optics, can mimic any surface curvature without additional fabrication difficulty, and are suitable for flat-top optics. The conventional metasurface design approach relies on imparting the required phase using meta-atoms and encounters challenges in amplitude modulation due to near-field coupling and varying transmittances among meta-atoms with different phases, making the design of flat-top beam shapers difficult. In this paper, we propose a complex-amplitude constraint Gerchberg–Saxton algorithm for designing a collimated flat-top beam shaper metasurface doublet, which avoids the influence of strong near-field coupling on the amplitude distribution and simultaneously considers the amplitude-phase characteristics of the meta-atoms. A collimated flat-top beam with exceptional homogeneity *U*_*p*_ of approximately 0.01, a wavefront error less than 0.1*λ*, and a transmittance higher than 86 % is experimentally obtained, comparable to commercial products based on freeform lenses. A collimated flat-top beam shaper metasurface doublet for generating flat-top beam is introduced, promoting efficient integration with laser systems.

## 1 Introduction

The beam shape plays a crucial role in laser applications, similar to the wavelength, pulse width and power. There is a high demand for a collimated flat-top beam that offers a constant and homogeneous irradiance profile within a certain range, which is essential for applications such as laser micromachining and laser ablation [1], [2], [3].

Beam homogenization and field mapping are two commonly used techniques to obtain a uniform beam. Beam homogenizers function by splitting the input beam into smaller beamlets, which are then overlapped in the output plane to form the desired shape. Typically, this setup comprises a lenslet array and a main lens. While such a configuration reduces spatial coherence, it is challenged to attain a uniform flat-top beam profile. On the other hand, field mappers utilize phase elements to morph discrete laser beams into a uniform beam. Depending on the number of phase elements used by the field mapper, this technique can be divided into two categories: focused beam shaping and cascaded beam shaping (the Schematic is given in Supplementary S_I_). The focused beam shaper utilizes a single phase element, such as diffractive optical elements (DOEs) or a spatial light modulator (SLM), which can only produce a flat-top beam in a specifically designed plane. This limitation requires precise positioning of the field mapper due to the non-planar nature of its emitted wavefront. In contrast, a cascaded beam shaper operates differently. Its first element introduces significant aberration to the wavefront, where the aberration can vary up to 20*λ* in phase, to restructure the irradiance of the beam after the wavefront has propagated across a specified distance. Subsequently, a second element of the beam shaper is designed with contours that effectively restore the output wavefront to a plane wavefront. This cascaded approach enables the output of a flat-top beam over a longer working length, overcoming the precise distance limitation. Moreover, this approach maintains the spatial coherence of the beam, making it a more advantageous solution than competing approaches. The conventional collimated flat-top beam shaper is mainly realized by freeform surfaces. Currently, several challenges remain, including the complexities of small-diameter freeform surface fabrication and integration difficulties [4], [5], [6].

Metasurfaces as planar optical components, exhibit versatile electromagnetic manipulation capabilities and can mimic any surface curvature without additional fabrication difficulty by imparting a spatially dependent phase delay using meta-atoms. The emergence of cascaded metasurfaces has significantly broadened the scope and flexibility of optical field manipulation, thereby greatly enhancing the suitability for realizing collimated flat-top beam shapers [7]–[15]. The conventional metasurface design approach imparting the required phase with meta-atoms assumes periodic boundary conditions for each element, which is inaccurate in most cases due to near-field coupling between elements when an element is surrounded by nonidentical structures [16], [17], [18]. In particular, the amplitude deviation caused by strong coupling will make the real amplitude distribution significantly deviate from the expected. Additionally, differences in transmission among meta-atoms with varying phases will impact the flat-top beam irradiance profile and transmittance. Hence, the conventional design approach fails to achieve a flat-top beam shaper based on metasurfaces.

In this paper, we proposed a complex-amplitude constraint Gerchberg–Saxton (GS) algorithm for designing a collimated flat-top beam shaper based on a metasurface doublet. We rewrap the phase to move the location of meta-atoms with a drastic diameter deviation to the edge of the beam with lower intensity to avoid the amplitude deviation caused by strong near-field coupling. In addition, we establish a relationship between the transmission and phase of meta-atoms to modulate the complex-amplitude constraints to mitigate the impact of varying transmission of meta-atoms on the flat-top beam irradiance profile. With this approach, we design a cylindrical collimated beam shaper metasurface doublet with excellent beam uniformity and verify it through full electromagnetic simulation and experiment. A flat-top beam irradiance profile with a homogeneity of *U*_*p*_ = 0.011, a wavefront error *PV* is less than 0.1*λ* and a high transmission of 86.57 % is experimentally obtained, which is comparable to that of commercial products based on freeform lenses. The collimated flat-top beam shaper metasurface doublet proposed in this paper will promote the integration of laser systems.

## 2 Design and simulation

A cylindrical collimated flat-top beam shaper based on a metasurface doublet is designed for verification in this paper.

With the conventional metasurface design approach, the near-field coupling between meta-atoms will cause the real amplitude and phase to deviate from the ideal values when meta-atoms designed under periodic boundaries are arranged aperiodically [19], [20], [21]. There is a violent amplitude oscillation when the size of meta-atoms drastically changes, usually at the phase wrapping point where the phase changes between the two values of 0 and 2*π*. A weak amplitude fluctuation occurs when the size of the elements smoothly changes. (The analysis process can be found in Supplementary S_II_.) Therefore, controlling the phase distribution will reduce the disturbance of the flat-top beam irradiance profile caused by near-field coupling. Ray tracing is the conventional collimated flat-top beam shaper design approach, which assumes that the transmission at coordinates on the surface is evenly equal to 1 [5], [22]. However, meta-atoms with different transmission phases often have different transmittances, which will directly lead to deviations from the ideal flat-top profile and a decrease in the transmittance. (Detailed comparisons are given in Supplementary S_III_.) Therefore, obtaining flat-top beam shaper with great uniformity using the conventional metasurface design approach is difficult.

**Figure 1:**
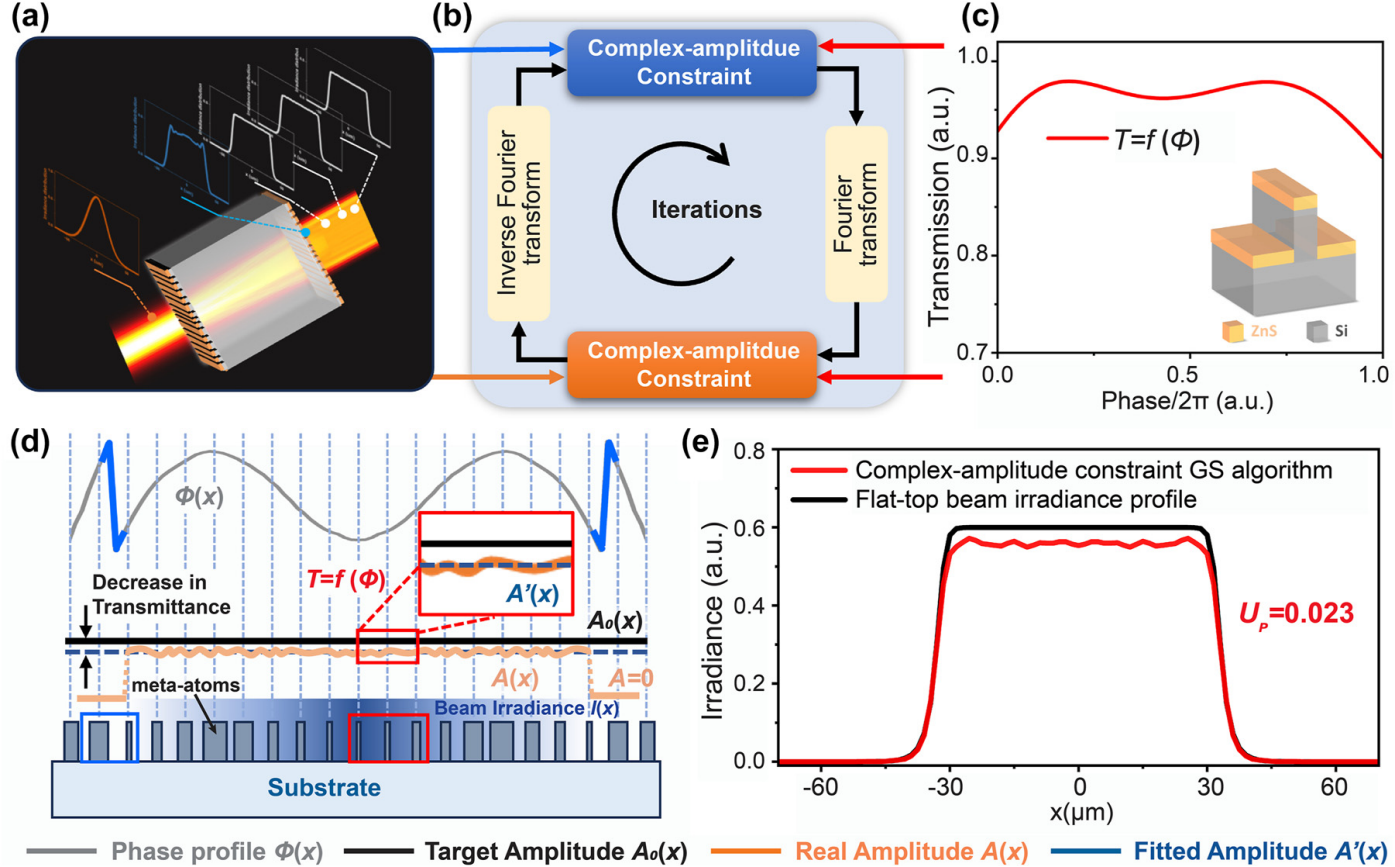
Design diagram of a collimated flat-top beam shaper metasurface doublet based on the complex-amplitude constraint GS algorithm. (a), (b) and (c) Reveal the design approach for the collimated flat-top beam shaper based on the metasurface doublet. (a) Shows a schematic of the cylindrical collimated flat-top beam shaper metasurface doublet. The input and output amplitudes are shown. (b) Shows the iternations of the algorithm to retrieve the phase of the metasurface doublet. The relationship of the transmission with the phase of the meta-atoms is shown in (c). (d) Establishes the main idea of the phase and amplitude constraints. (e) Shows a flat-top beam irradiance profile with a great homogeneity.

Thus, a complex-amplitude constraint GS algorithm for collimated flat-top beam shaper metasurface doublet design is proposed in this paper. A detailed diagram of the algorithm is shown in Figure 1. A schematic of the collimated flat-top beam shaper metasurface doublet is shown in Figure 1(a). An incident beam with a Gaussian irradiance profile is shaped into a collimated flat-top beam within a certain range. The cylindrical collimated flat-top beam shaper metasurface doublet is designed on both sides of a Si substrate. A TM-polarized Gaussian beam with a wavelength of 5 μm and a waist radius of 32 μm is shaped into a flat-top beam with a radius of 32 μm. The meta-atoms form a one-dimensional silicon grating coated with a zinc sulfide film with a period of 1.5 μm. The height of the Si grating is 4.53 μm, and the thickness of the ZnS film deposited on top of the Si grating is 1.33 μm. Full phase coverage (0–2*π*) with high transmission (>90.0 %) is obtained. The distribution of the transmission versus phase is shown in Figure 1(c). The thickness of the substrate is 130 μm.

**Figure 2:**
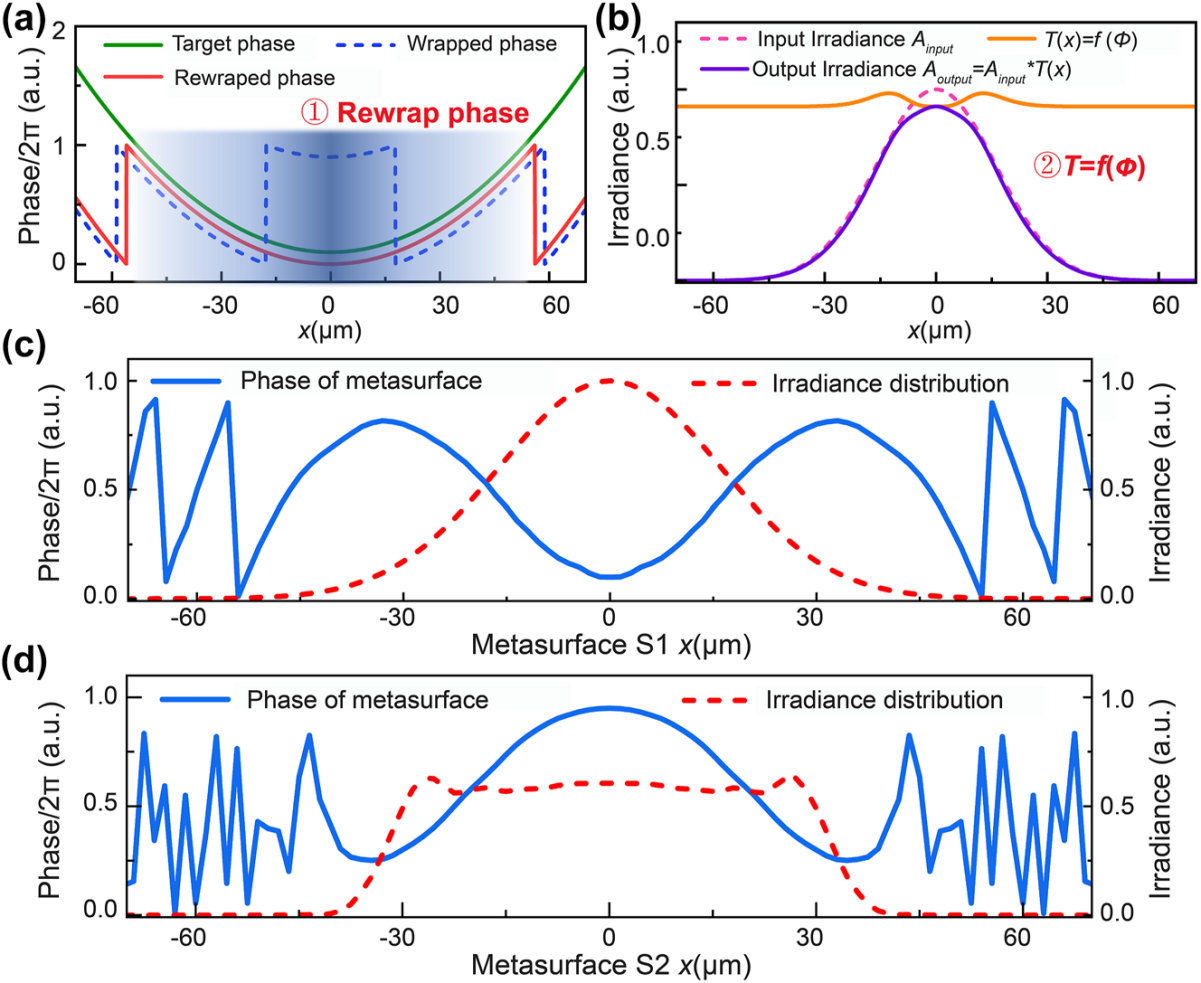
Constraints of the complex-amplitude GS algorithm and converged phase of the designed cylindrical collimated flat-top beam shaper metasurface doublet. (a) and (b) Establish the specific operations of phase and amplitude modulation. (c) Phase of metasurface S1 with Gaussian beam incidence. (d) Phase of metasurface S2 with a flat-top beam output.

This complex-amplitude constraint GS algorithm is a development of the conventional GS algorithm [23], [24], [25]. In this algorithm, we rewrap the phase distribution to move coordinates with strong near-field coupling out to the edge of the beam with lower energy and make the electromagnetic transmission modulation properties of meta-atoms as a function of the phase in each iteration, as revealed in Figure 1(d). A flat-top beam with a more uniform irradiance profile is obtained in Figure 1(e). In more detail, a beam with a Gaussian irradiance profile and a plane wavefront incident on metasurface S1 is the input, as shown by the red line in Figure 1(a). Metasurface S1 can be seen as a plate to introduce a phase difference, and the phase and amplitude change during iteration. To avoid violent amplitude deviations in the area with strong near-field coupling, the phase is rewrapped to move the phase jump point to the edge of the beam with lower irradiance in each iteration and then retained in the next iteration, *ϕ*_S1_kth_ = Rewrap(*ϕ*_object_kth_ − *ϕ*_plane_) a schematic of the phase modulation is shown in Figure 2(a). With the correlation between the transmission and phase of *T* = *f*(*ϕ*), the transmission of metasurface S1 *T*_S1_kth_ = *f*(*ϕ*_S1_kth_). The input amplitude constraints in each iteration on the object plane are replaced by *A*_Gaussian_ ⋅ *T*_S1_kth_, as shown in Figure 2(b). Then, Fourier transform to the image plane is applied.

The phase and amplitude after metasurface S2 are used as the output of the iterations, which are calculated by inverse Fourier transform of the ideal flat-top beam at metasurface S2, as shown by the blue line in Figure 1(a). With the same approach, the phase and amplitude constraints of metasurface S2 are *ϕ*_S2_kth_ = Rewrap(−*ϕ*_out_ − *ϕ*_image_kth_) and *T*_S2_kth_ = *f*(*ϕ*_S2_kth_), and the input amplitude of the image surface is replaced by *A*_out_ ⋅ *T*_S2_kth_. Then, inverse Fourier transform to the object plane is applied.

Iterations are performed until convergence, and the final phase distribution of metasurfaces S1 and S2 can be obtained, with which a collimated flat-top beam will be output. The retrieved phase distributions of the two metasurfaces are depicted in Figure 2(c) and (d). The blue solid lines present the phase distribution of the metasurfaces, and the red dashed lines present the irradiance radiated onto the metasurfaces. The coordinates of the phase jump between the values of 0 and 2*π* are at approximately 50 μm–60 μm, where the intensity is nearly zero, consistent with the idea of the complex-amplitude constraint GS algorithm proposed in this paper. (More detailed steps of the iteration and the convergence of the algorithm are given in Supplementary S_IV_.)

**Figure 3:**
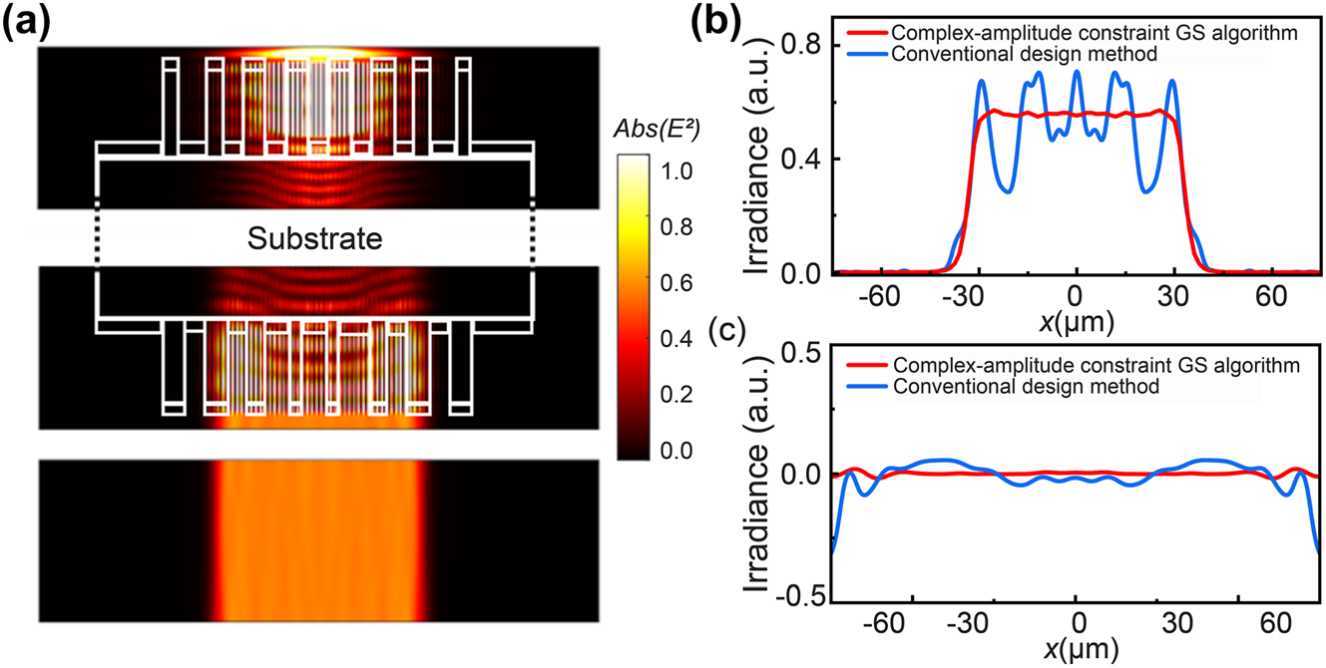
Compact cylinder cascaded beam shaper doublet designed in this paper. (a) The distribution of electric field through the cascaded beam shaper, only areas of metasurface S1, metasurface S2 and output flat-top beam is shown. And the white line approximates the outline of metasurfaces and substrate, (b) the flat-top irradiance distribution of the flat-top profile, ideal design (no effects of near-field coupling) and real design (simulated by FDTD), (c) the flat-top phase distribution of the plane wave, ideal design and real design (simulated by FDTD).

Finite difference time domain (FDTD) simulations are employed to validate the designed collimated flat-top beam shaper metasurface doublet. The electric field distribution within the collimated flat-top beam shaper metasurface doublet is presented in Figure 3(a). Three representative areas around metasurface S1, around metasurface S2 and the output flat-top beam are shown. The results show that a collimated flat-top beam irradiance profile can be obtained in a certain range. As displayed in Figure 3(b), the homogeneity *U*_*p*_ of the flat-top beam irradiance profile achieved is improved from 0.682 to 0.023, the RMS is less than 1.02 %, and the transmission is as high as 93.25 % (the plateau uniformity *U*_*p*_ and beam uniformity RMS are specified by ISO 13694 [26], [27]), which are comparable to commercial products. As a main feature of the collimated beam shaper, the output wavefront is nearly a plane wave with a wavefront error *PV* less than 0.01*λ*, as indicated in Figure 3(c). The reason for the small difference from the simulated results with an ideal flat-top beam is mainly due to the weak coupling when the size of meta-atoms smoothly changes, which makes precise modulation difficult.

A higher transmittance can be achieved with higher efficiency meta-atoms. In addition, the effectiveness of using this method to obtain a circular flat-top beam has also been verified; details can be found in Supplementary S_V_.

## 3 Fabrication and characterization

To experimentally confirm the collimated flat-top beam shaper based on metasurfaces, two 3.6 mm × 3 mm cascaded metasurfaces to shape a TM-polarized Gaussian beam with a waist radius of 600 μm into a flat-top beam with the same radius were designed and fabricated. To reduce the difficulty of manufacturing, the two cascaded metasurfaces were fabricated on two silicon substrates with thicknesses of 0.5 mm with air in between. The metasurfaces were fabricated using electron beam lithography and dry etching manufacturing techniques. Please refer to the Methods section for the fabrication process.

**Figure 4:**
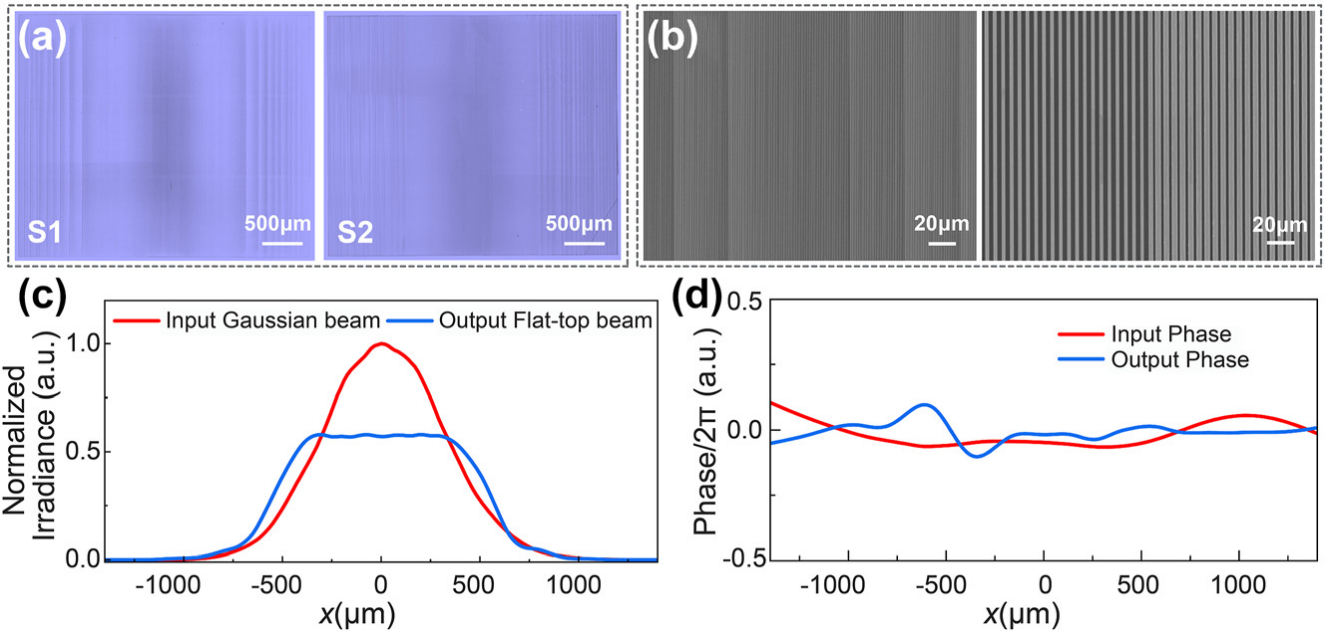
Experimental results for the cylindrical collimated flat-top beam shaper based on cascaded metasurfaces. (a) Optical microscopy images of metasurfaces S1 and S2. (b) SEM images depicting the metasurfaces. (c) Detected normalized irradiance beam profile of the input Gaussian beam and output flat-top beam. (d) Phase distribution of the input and output beams reconstructed by the TIE method.

Images of metasurfaces S1 and S2 under an optical microscope are presented in Figure 4(a). Considering that the samples target cylindrical shaping devices, their unit structures feature uniform vertical alignment and inherent left–right symmetry. Scanning electron microscopy (SEM) images of the metasurfaces are shown in Figure 4(b). The locations of rapid phase transitions correspond to areas with left-right symmetry and high brightness. The energy distributions of the input Gaussian beam and output flat top beam are detected, as shown in Figure 4(c). The homogeneity of the flat-top irradiance profile is *U*_*p*_ = 0.011, the RMS is less than 1.34 %, and the transmission is as high as 86.57 %. The wavefronts of the input beam and the output collimated flat-top beam are also shown in Figure 4(d), which are retrieved by the transport of intensity equation (TIE) method [28]. The wavefront error PV of the collimated flat-top beam is less than 0.1*λ*. More detail of the characterization system can be found in the methods. Compared to expectations, the side of the flat-top beam profile is not as sharp as the ideal and the phases are asymmetric, mainly due to fabrication errors. (More analysis is provided in Supplementary S_VI_.)

## 4 Conclusions

In conclusion, we have proposed a complex-amplitude constraint GS algorithm to design a collimated flat-top beam shaper metasurface doublet. With this approach, we mitigate the influence of violent amplitude oscillations at phase wrapping points and variations in meta-atom transmission on the flat-top beam irradiance. The collimated flat-top beam shaper metasurface doublet is designed with multilayer grating meta-atoms, achieving excellent plateau uniformity in the flat-top beam profile and transmission characteristics, which is verified by experiments. Our work provides a paradigm for a collimated flat-top beam shaper based on a metasurface doublet to shape a Gaussian beam into a flat-top beam. It is expected to facilitate integration with laser systems to achieve miniaturization and high performance.

## 5 Methods

### 5.1 Design and simulation

The collimated flat-top beam shaper metasurface doublet is realized by establishing a multilayer grating meta-atom library. We optimize and sweep the amplitude and phase of meta-atoms using rigorous wave coupled analysis and particle swarm optimization. The collimated flat-top beam shaper metasurface doublet is simulated by commercial lumerical FDTD software.

### 5.2 Fabrication

Two cascaded metasurfaces are fabricated on two independent silicon substrates. Antireflection films consisting of germanium and zinc sulfide are deposited on the back side. The substrate is first spin-coated with a PMMA photoresist and baked. Then, the metasurfaces are patterned in the photoresist via an electron beam lithography (EBPG5200, Raith) system and developed in a mixed solution of MIBK and IPA. After development, a chromium film is deposited and soaked in remover for more than 2 h to obtain the hard mask. Finally, inductively coupled plasma reactive ion etching (ICP-RIE) with a mixture of SF_6_ and CHF_3_ is applied to etch the silicon. Finally, the ZnS layer is deposited.

### 5.3 Characterization

The homogeneity and intensity of the beam after shaping are characterized. Two lenses are used to scale the Gaussian beam waist from 6 mm to 0.6 mm as input. The metasurfaces are aligned by a motorized stage with a minimum incremental movement of 0.05 μm. The output beam irradiance profile is detected by an infrared camera. A photograph of the characterization system can be found in the Supplementary S_VI_.

## Supplementary Material

Supplementary Material Details
