# Supplementary material for: Collimated flat-top beam shaper metasurface doublet based on the complex-amplitude constraint Gerchberg–Saxton algorithm
Source: Nanophotonics. 2024 Jan 11;13(8):1379–85. doi: 10.1515/nanoph-2023-0719 (PMC11636518; doi:10.1515/nanoph-2023-0719)
Supplement: Supplementary file 1 — Supplementary Material Details [file j_nanoph-2023-0719_suppl_001.pdf]

Dongbai Xue†,Xiong Dun†,Zeyong Wei†,DongDong Li,Jingyuan Zhu,Zhanyi Zhang,Zhanshan Wang,Xinbin Cheng*

Supplementary material: Collimated flat-top beam shaper metasurface doublet based on the complex-amplitude constraint Gerchberg-Saxton algorithm

Contents

S_I. Schematic of focused and collimated beam shapers

S_{II}. Influence of near-field coupling on the phase and amplitude

S_{III}. Flat-top beam profile designed without utilizing the approach proposed in this paper

S_{IV}. Detailed steps and convergence of the complex-amplitude constraint Gerchberg-Saxton algorithm

S_V. Validation of the circular flat-top beam shaper metasurface doublet designed with the approach proposed in this paper

S_{VI}. Experimental characterization

Dongbai Xue†,Xiong Dun†,Zeyong Wei†,DongDong Li,Jingyuan Zhu,Zhanyi Zhang,Zhanshan Wang,Xinbin Cheng*, Institute of Precision Optical Engineering, School of Physics Science and Engineering, Tongji University, Shanghai 200092, China; MOE Key Laboratory of Advanced Micro-Structured Materials, Shanghai 200092, China; Shanghai Frontiers Science Center of Digital Optics, Shanghai 200092, China; Shanghai Professional Technical Service Platform for Full-Spectrum and High-Performance Optical Thin Film Devices and Applications, Shanghai 200092, China; Shanghai Institute of Intelligent Science and Technology, Tongji University, Shanghai 200092, China.

S_I. Schematic of focused and collimated beam shapers.

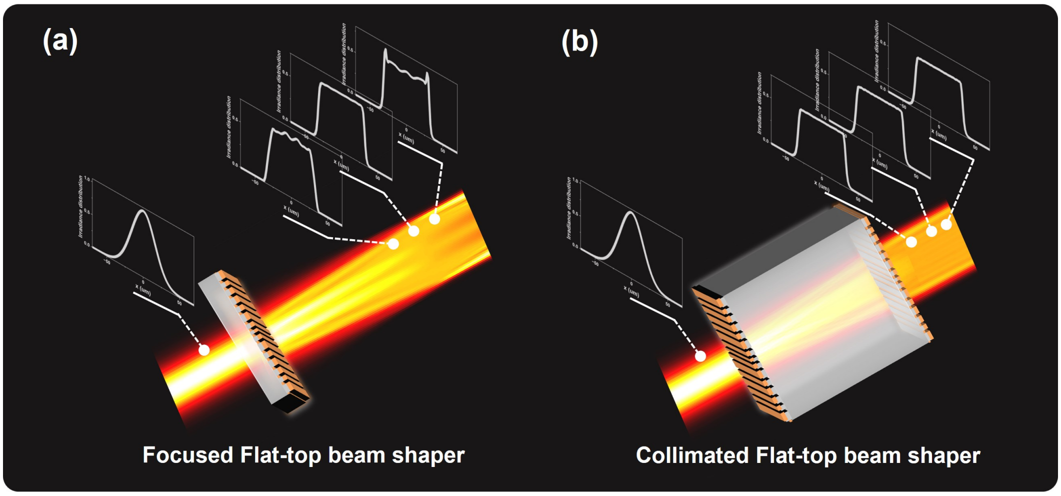

Fig. S1: Schematic of focused and collimated beam shapers.

S_{II}. Influence of near-field coupling on the phase and amplitude.

Near-field coupling will affect the distribution of the amplitude and phase of a meta-atom surrounded by nonidentical meta-atoms. The study of the metasurface amplitude deviation laws aims at achieving precise amplitude control. The amplitude and phase distributions of plane waves propagating through a metasurface are depicted in Fig. S1. The blue lines correspond to the simulated phase and amplitude detected by the FDTD power monitor, and the red lines depict the ideal phase and amplitude assuming neglect of the near-field coupling effect. The distance between the monitor and metasurface is more than three wavelengths, which is large enough to be consider as far field [1]. Strong near-field coupling with significant amplitude oscillations occurs when meta-atoms drastically change in size, typically at the phase wrapping point, as indicated in the red area in Fig. S1(a) and (b). Weak coupling with minor amplitude fluctuations occurs where the size of meta-atoms smoothly changes. Due to spatial sampling limitations and near-field coupling effects, a notable disparity exists between the actual and ideal amplitude and phase distributions.

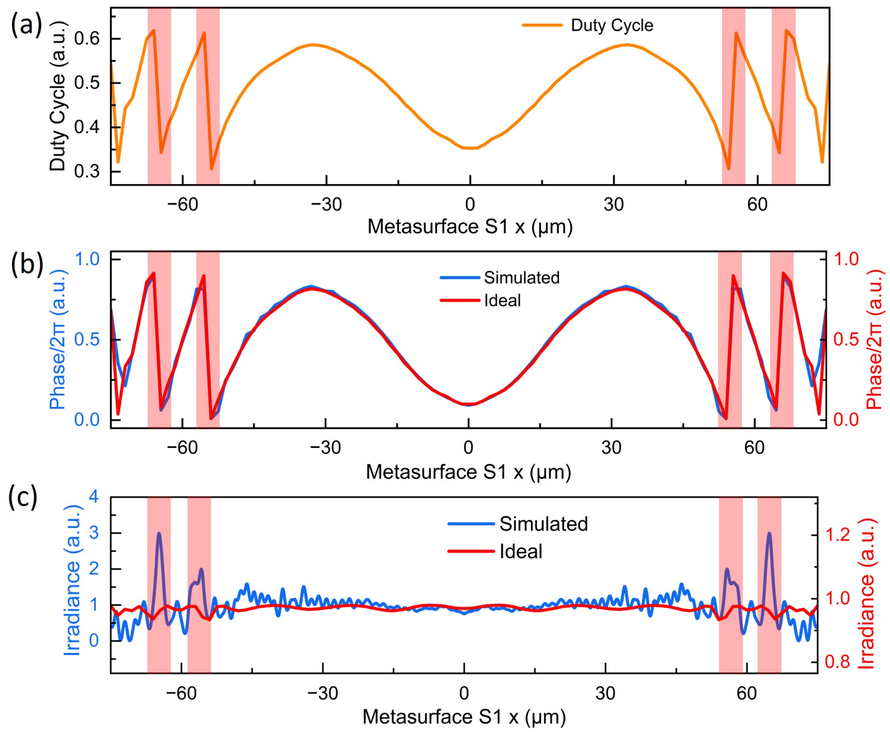

Fig. S2: Simulated and ideal phase and amplitude of metasurface S1 in the designed collimated flat-top beam shaper metasurface doublet.

S_{III}. Flat-top beam profile designed without utilizing the approach proposed in this paper.

The conventional metasurface design method cannot achieve the desired flat-top beam profile with great homogeneity. As verification, the simulation results of the flat-top beam irradiance profile for the metasurface doublet obtained using the conventional design method are shown in Fig. S2. The flat-top beam irradiance profile is characterized by a homogeneity of $U_p = 0.682$, an RMS of approximately 26.5%, and a wavefront error PV of approximately 0.38λ.

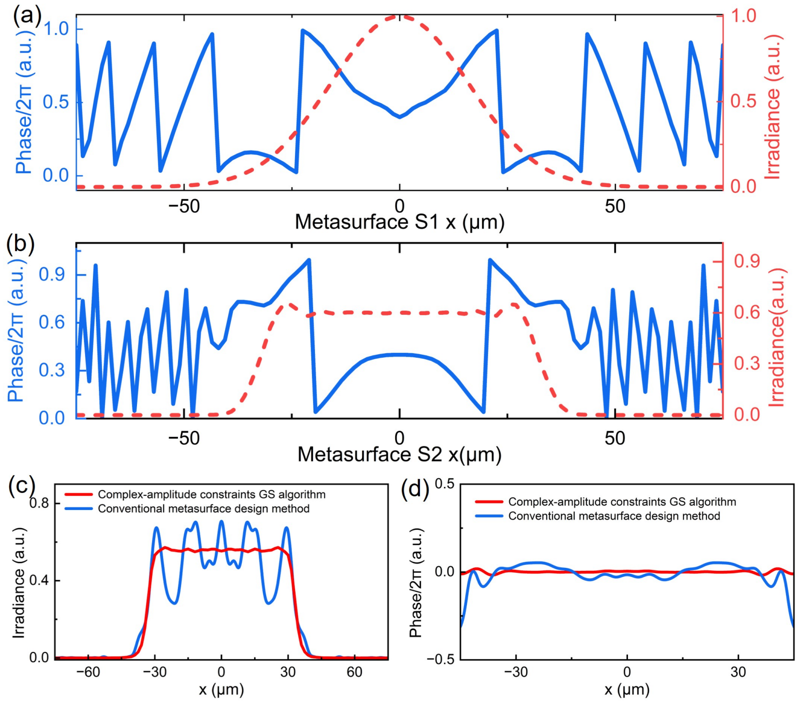

Fig. S3: Flat-top beam profile obtained with the conventional design method.

When the correlation between the amplitude and phase of meta-atoms is not considered, only strong near-field coupling is avoided. The designed phases of the two cascaded metasurfaces are shown in Fig. S3(a)(b). The irradiance distribution and phase of the output beam are shown in Fig. S3(c)(d). The results show that the phase difference with the ideal plane wave PV is less than 0.01λ, which meets our expectations; however, the irradiance profile deviates from the flat-top profile, and U_p=0.078 is more than three times larger than that of the profile designed with the complex-amplitude-modulated approach proposed in this paper.

When the phase is not rewrapped during the iterations of the GS phase retrieval and only the amplitude and phase of meta-atoms are introduced, the homogeneity of the flat-top profile will be seriously affected. The results show that the irradiance distribution and phase both deviate from the ideal, as shown in Fig.S4. The homogeneity

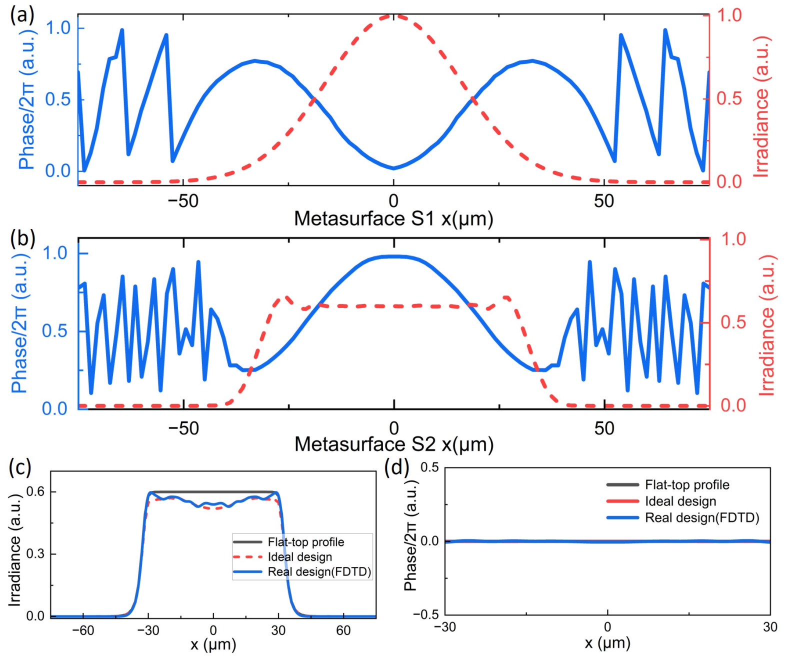

Fig. S4: Flat-top profile designed with strong near-field coupling avoided.

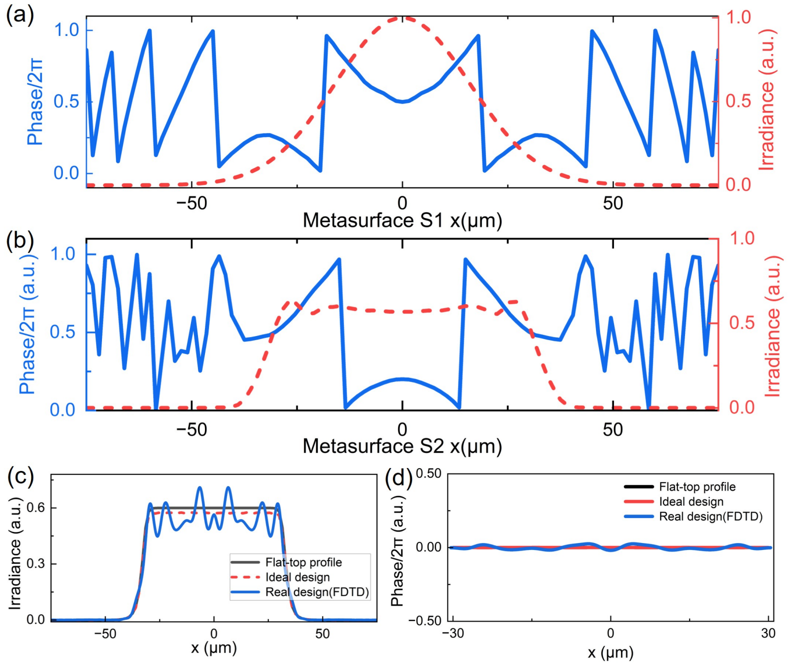

Fig. S5: Flat-top profile when the amplitude and phase of meta-atoms are introduced.

of the flat-top profile U_P=0.236, and the phase difference with the plane wave PV is 0.05λ.

S_{IV}. Detailed steps and convergence of the complex-amplitude constraint Gerchberg-Saxton algorithm

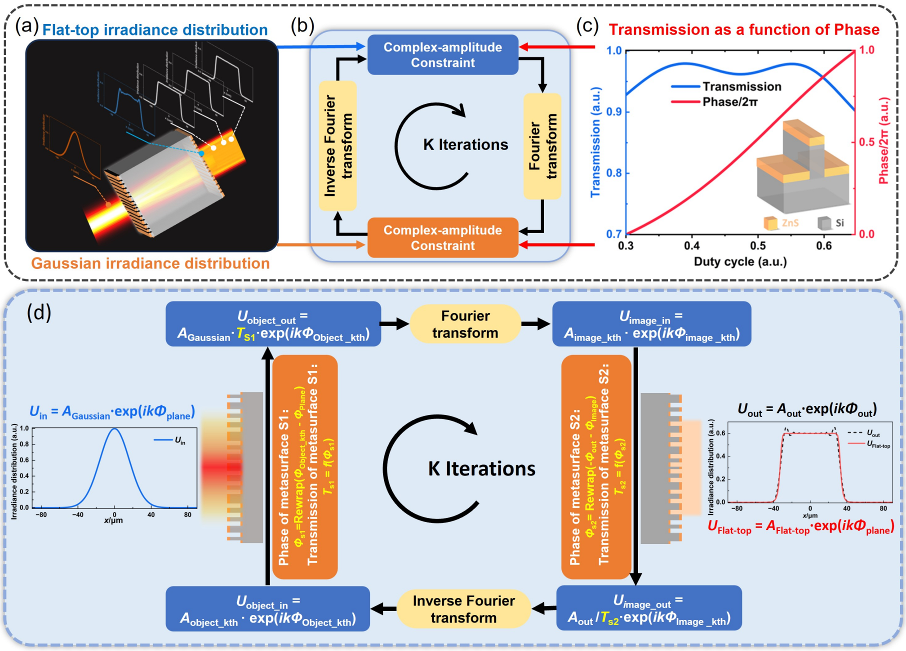

Fig. S6: Design diagram of a collimated flat-top beam shaper metasurface doublet. (a) Schematic of the collimated flat-top beam shaper metasurface doublet. (b) Complex-amplitue constraint GS algorithm to retrieve the phase. (c) Transmission and phase of meta-atoms. (d) is a detailed version of (b), establishing the specific steps of the complex-amplitude constraint GS algorithm.

The complex-amplitude constraint GS algorithm design approach of a collimated flat-top beam shaper metasurface doublet can be divided into the following steps:

Step 1: The iterative approach in this paper starts from a random phase as conventional. Metasurface S1 at the object plane can be seen as a plate with uniform amplitude and phase when just starting, $T_{S1\_1th} = 1$. Thus, the original amplitude in the first iterations is $A_{object\_1th} = A_{Gaussian} * T_{S1\_1th}$, and then, Fourier transform to the image plane is applied.

Step 2: The transmission T_{S2_kth} and phase ϕ_{S2_kth} of metasurface S2 , which can be seen as a plate to compensate for the phase difference between ϕ_{out} and ϕ_{image}, are retrieved. Here, ϕ_{out} and A_{out} refer to the phase and amplitude output from metasurface S2, which are calculated by inverse Fourier transform of the flat-top beam $A_{flat\text{-}top}$ to the output plane of metasurface S2. To avoid violent amplitude fluctuations in the area with strong near-field coupling, the phase is rewrapped to move the phase jump point to the edge of the incident beam with lower energy in each iteration, $\phi_{S2\_kth} = Rewrap(-\phi_{out} - \phi_{image\_kth})$. T_{S2_kth} is the transmission of metasurface S2, which is correlated with the phase of S2. The relationship can be confirmed by correlating the transmission and phase of meta-atoms, regarded as a function of $T_{S2\_kth} = f(\phi_{S2\_kth})$, as shown in Fig. 1(c).

Step 3: The phase ϕ_{image} is retained, and the amplitude Aimage at the image plane is substituted by the object plane amplitude $A_{\mathrm{S2\_kth}} = A_{\mathrm{out}}/T_{\mathrm{S2\_kth}}$. Then, inverse Fourier transform to the object plane is applied.

Step 4: The amplitude and phase of metasurface S1 are obtained as in **Step 2**, $T_{\mathrm{S1\_kth}} = f(\phi_{\mathrm{S1\_kth}})$ and $A_{\mathrm{S1\_kth}} = A_{\mathrm{Gaussian}} * T_{\mathrm{S1\_kth}}$.

This process is repeated from **Step 1**. The final converged phase distribution of metasurfaces S1 and S2 can be obtained, with which a flat-top beam will be output after the metasurface doublet.

Convergence of the complex-amplitude constraint GS algorithm

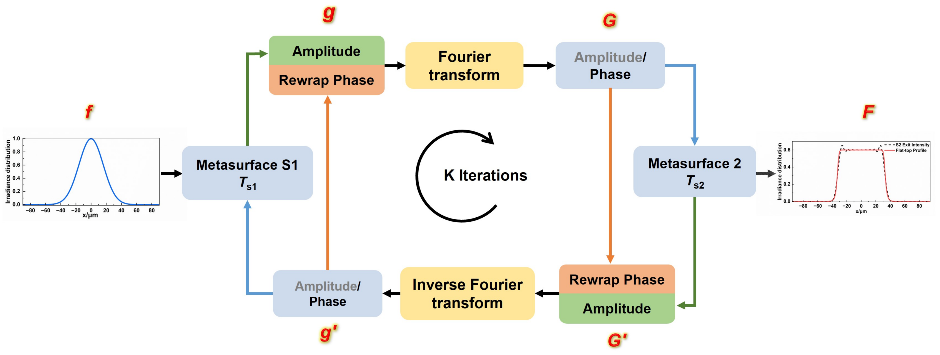

Fig. S7: Iterations of the modified GS algorithm.

The iterations of the complex-amplitude constraint GS algorithm are shown in Fig. S5. f and F are the electric fields of the Gaussian beam and flat-top beam, respectively. G and ϕ are the electric field and phase in the Fourier domain, and g and are the electric field and phase in the object domain. T_{S1} and T_{S2} are the spatial distributions of the transmittance of metasurface S1 and metasurface S2, respectively. The iterations can be represented as Eqs. S1-S4:

$$G_k(u) = |G_k(u)| \exp[i\phi_k(u)] \tag{S1}$$

$$G_k^{'}(u) = |\frac{F(u)}{T_{S2,k}}| \exp[i\phi_k(u)] \tag{S2}$$

$$g_k^{'}(x) = |g_k^{'}(x)| \exp\left[i\theta_k^{'}(x)\right] \tag{S3}$$

$$g_{k+1}(x) = |f(x) \cdot T_{S1,k}| \exp[i\theta_{k+1}(x)] = |f(x) \cdot T_{S1,k}| \exp\left[i\theta_k^{'}(x)\right] \tag{S4}$$

The convergence of the algorithm can be monitored by computing the squared error. Since $G_K^{'}(x)$ is formed from $G_K(x)$ by making the minimum changes to satisfy the Fourier domain constraints, the squared error can be expressed as Eq. S5 according to Parseval's theorem,

$$E_{Fk}^2 = N^{-2} \sum_u \left|G_k(u) - G_k^{'}(u)\right| = \sum_x \left|g_k(x) - g_k^{'}(x)\right|^2 \tag{S5}$$

Similarly, for the error reduction algorithm, the squared error in the object domain can be expressed as

$$E_{ok}^2 = \sum_x |g_{k+1}(x) - g_k^{'}(x)|2 = N^{-2} \sum_u \left|G_{k+1}(u) - G_k^{'}(u)\right|^2 \tag{S6}$$

Comparing the two equations at all points x,

$$\left|g_k(x) - g_k^{'}(x)\right|^2 \geq \left|g_{k+1}(x) \cdot \frac{T_{s1,k}}{T_{s1,k+1}} - g_k^{'}(x)\right|^2 \tag{S7}$$

Therefore,

$$E_{Fk}^2 \geq E_{ok}^2 \tag{S8}$$

Similarly,

$$\left|G_{k+1}(u) - G_k^{'}(u)\right|^2 == \left|G_{k+1}(u) - G_{k+1}^{'}(u) \cdot \frac{T_{s2,k+1}}{T_{s2,k}}\right|^2 \tag{S9}$$

$$E_{ok}^2 \geq E_{F,k+1}^2 \tag{S10}$$

Combining Eq. S8 and Eq. S10 gives the desired result

$$E_{F,k+1}^2 \leq E_{0k}^2 \leq E_{Fk}^2 \tag{S11}$$

That is, the error can only decrease in each iteration.

S_V. Validation of the circular flat-top beam shaper metasurface doublet designed with the approach proposed in this paper

With the approach proposed in this paper, an input TM-polarized circular Gaussian beam with a waist of 32 μm and a wavelength of 5 μm is shaped into a flat-top beam with a radius of 32 μm. A meta-atom period of 1.5 μm is selected, which is shown in Fig. S7(c). The height of the cylinder is 6.8 μm, and the thickness of the ZnS layer is 0.5 μm.

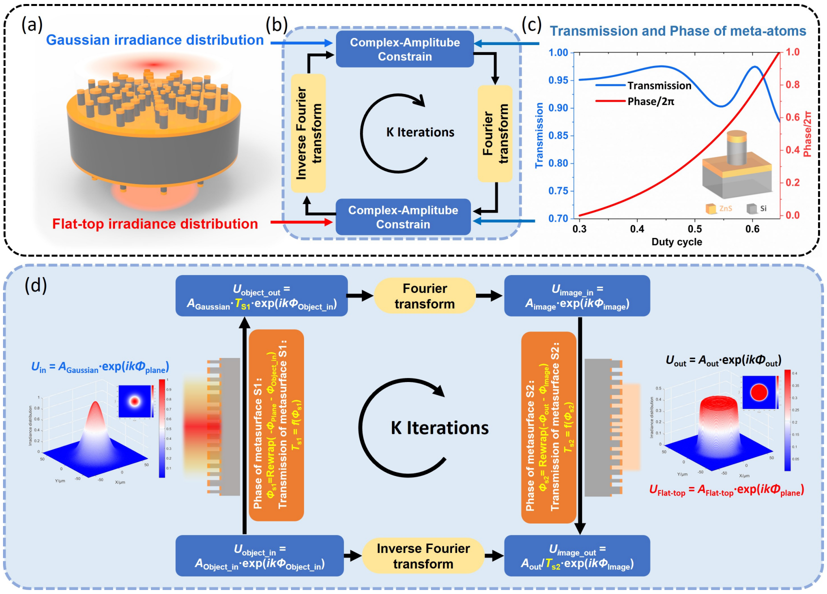

Fig. S8: Design diagram of the beam shaper based on the metasurface doublet.

Simulating the 3D metasurface doublet is not feasible due to computational limitations. To demonstrate the applicability of the beam shaper metasurface doublet design method, we simulate the amplitude and phase distribution of a plane wave passing through a single metasurface using the same method as in S_I. As shown in Fig. S8, the simulated and ideal amplitude and phase distributions are consistent with those in S_I. Therefore, this is sufficient to illustrate the applicability of the method to obtaining a circular flat-top beam.

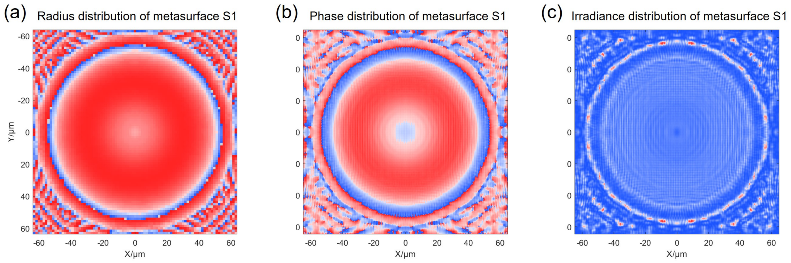

Fig. S9: Simulation results of the amplitude and phase distribution of the 3D metasurface.

S_{VI}. Experimental characterization

Two metasurface samples are illustrated in Fig. S9. (a) shows the appearance of the samples, (b) and (c) show the complete stitched sample image under the optical microscope, and (d-f) show the microstructures of local areas observed by SEM. Unfortunately, the etching depths of the silicon meta-atoms with different linewidths are different, and the thickness of the zinc sulfide film at the top and bottom of the meta-atoms is not uniform. These will influence the flat-top beam irradiance profile.

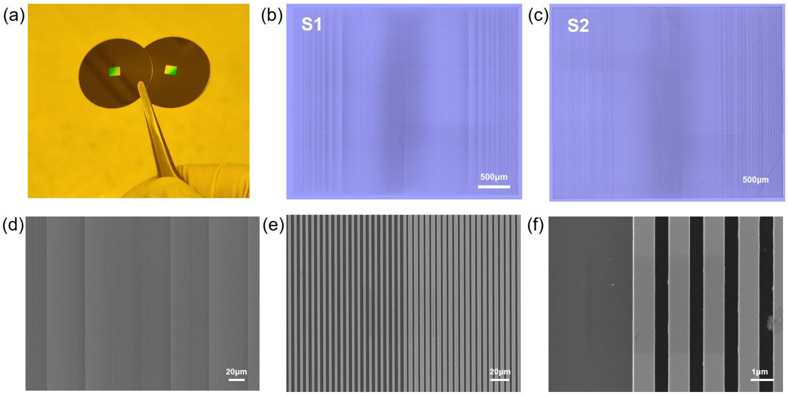

Fig. S10: Processed metasurface samples.

Fig. S10 depicts the optical system employed for characterizing the cascaded metasurface beam shaper. A laser with a wavelength of 5 μm is incident and scaled to a waist of approximately 0.6 mm after two zinc selenide lenses. The relative position and rotation of the metasurfaces are tuned by motorized stages and rotation mounts. The output beam is detected by an infrared camera.

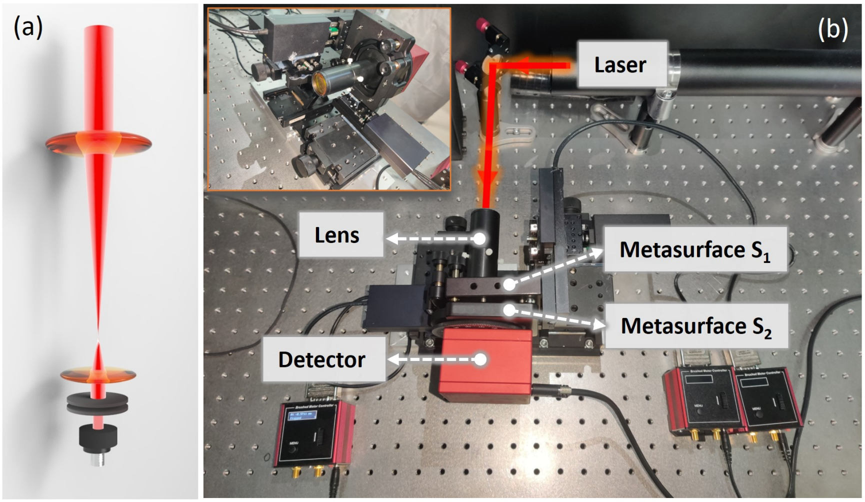

Fig. S11: Schematic of the experimental characterization system.

The flat-top beam profile I_0 is detected. To obtain the output beam wavefront with the TIE method [2], the beam irradiances I_1 and I_2 at distances of 3 mm and 6 mm from the I_0 face are detected, as shown in Fig. S11. A flat-top beam can evidently be obtained within a certain range from the exit surface, while at farther distances, it will diffract into Airy patterns.

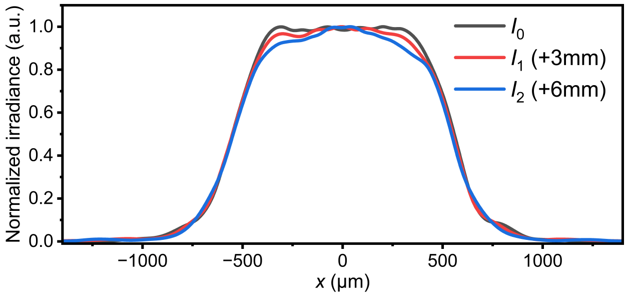

Fig. S12: Output beam irradiance distribution at different distances from the exit metasurface.

Although a flat-top beam irradiance profile is obtained, significant discrepancies are observed in the radius of the flat-top beam and the steepness of the sidewalls of the irradiance distribution, which deviate from the ideal design, as depicted in Fig. S12. These disparities primarily arise from manufacturing errors in the samples, as illustrated in Fig. S9(f).

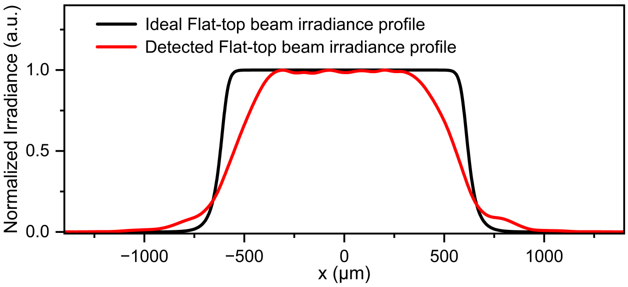

Fig. S13: Normalized irradiance distribution of the detected along with the ideal flat-top beam profile.

Analysis of system alignment requirements

The alignment requirements of these two metasurfaces are analyzed in this paper. These analyses played a crucial role in guiding our experimental adjustments. In this section, we provide a detailed explanation of the results from these analyses of alignment requirements. The results indicate that positional deviation of the laser is the most significant adjustment error affecting the output. However, it is the only factor that causes the energy profile of the output flat-top beam to tilt, thus it can be observed and specifically adjusted during the experiment. Angular deviations in the system do not significantly affect the output beam, and can be easily met through

mechanical adjustments. Alignment deviations between the two metasurfaces can be easily addressed with an electric motorized stage. Therefore, the misalignment of these two metasurfaces has a considerable tolerance, and can be specifically adjusted based on the irradiance distribution of the output beam.

When there are no alignment errors, the beam shaper follows the configuration shown in the Fig. S14. The TM-polarized light propagates from left to right and is shaped into a flat-top beam.

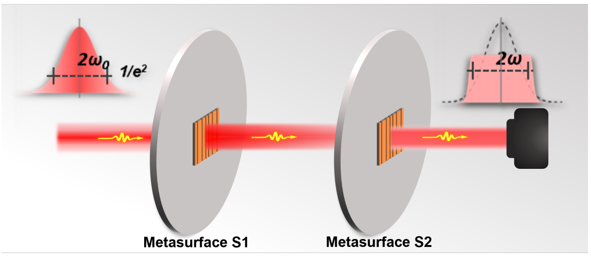

Fig. S14: The ideal configuration with no alignment errors.

This paper separately analyzes the impact of various factors on the beam shaping: the angular deviation of the laser, the alignment error between the first metasurface and the Gaussian beam, the spacing error between two cascaded metasurfaces, the alignment errors of the two metasurfaces, the rotational and yaw angle deviations of the metasurfaces, and the positional error of the detector image plane. The specific results are as follows:

(1) Laser angle deviation

The Gaussian beam incident on metasurface S1 may form an angle with the surface normal of the metasurface, rather than being ideally perpendicular. This tilt angle causes the beam center to deviate from metasurface S2 after passing through S1, affecting the flat-top profile of the output beam. As shown in Fig. S16 (a)-(c), there is a decrease in beam irradiance uniformity and a tendency for the beam to tilt. However, by moving metasurface S2 so that the central chief ray passes through the center of S2, the output flat-top beam profile can be excellently compensated. As shown in Fig. S16 (d)-(f), the beam irradiance uniformity is close to ideal, with no significant change in spot size, only a shift is displayed. Therefore, the system has a high tolerance for the angle of incidence.

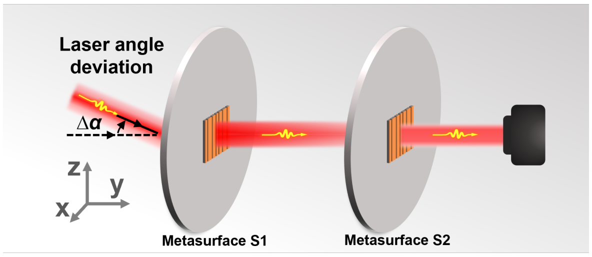

Fig. S15: Misalignment configuration resulting from Laser Angle Deviation.

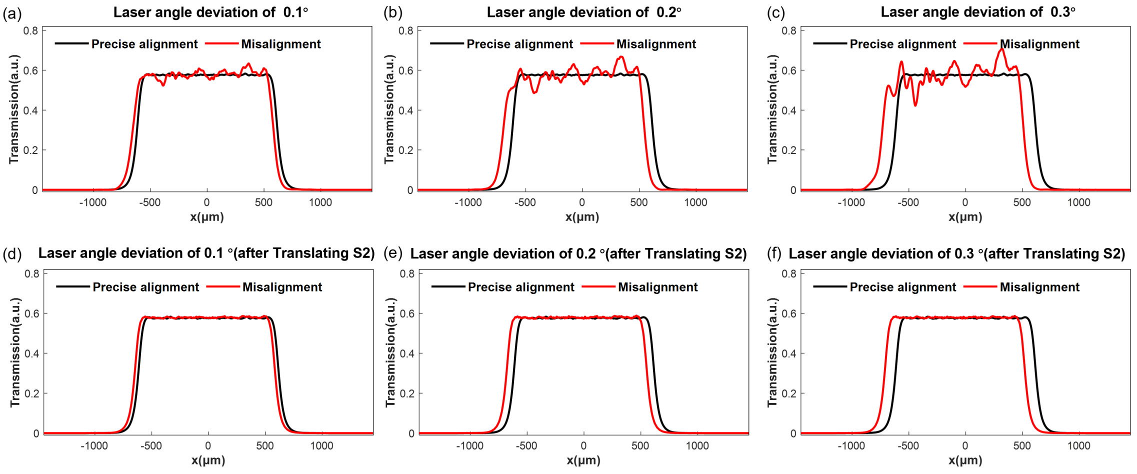

Fig. S16: Flat-top beam irradiance profile with Laser Angle Deviation and moving compensated.

(2) Beam position deviation

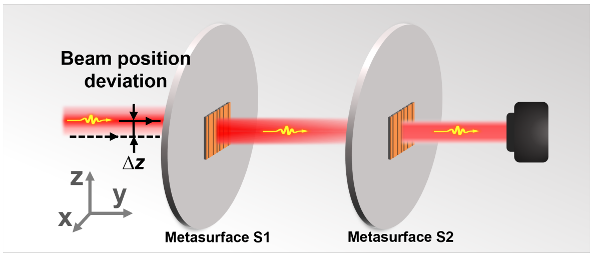

Fig. S17: Misalignment configuration resulting from Beam position deviation.

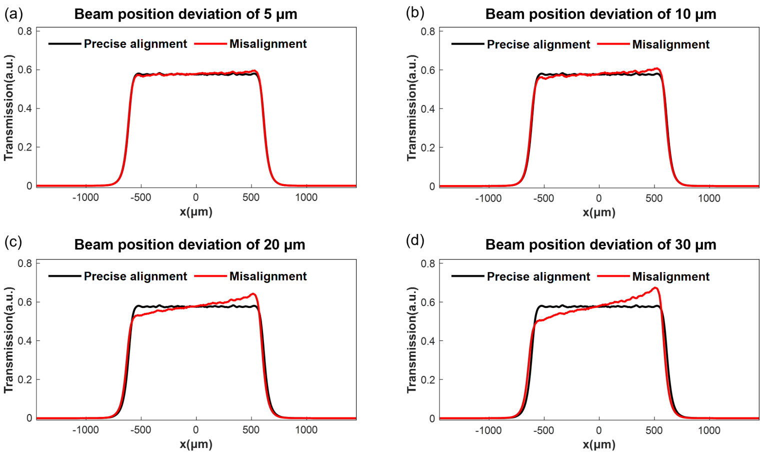

Fig. S18: Flat-top beam irradiance profile with Beam position deviation.

he Gaussian beam incident on metasurface S1 may have a positional deviation, meaning that the central chief ray of the incident light has an off-axis error relative to the center of the metasurface. As shown in Fig. S18, when the off-axis amount is less than $\pm 10\ \mu$m, the flat-top effect is close to the ideal outcome. As the deviation increases, it becomes difficult to eliminate the trend of tilted energy distribution by appropriately adjusting the position of metasurface S2. However, this factor has a distinct impact on the energy profile of the flat-top beam and is not caused by other factors, allowing for targeted adjustments during experiments.

(3) Metasurface Spacing Deviation

The two cascaded metasurfaces are fabricated on two silicon wafers in the experiment, leading to a spacing error between them. The ideal spacing is 12.2 mm, and when there is a spacing error within ± 0.3 mm, the output beam are shown in the Fig. S20, does not exhibit significant differences compared to the ideal flat-top. When the spacing error reaches ± 0.5 mm, the edge profile of the flat-top beam tends to evolve towards a super-Gaussian or inverse Gaussian beam, but the change is still minor. Therefore, the assembly tolerance for the spacing can be less than ± 0.5 mm.

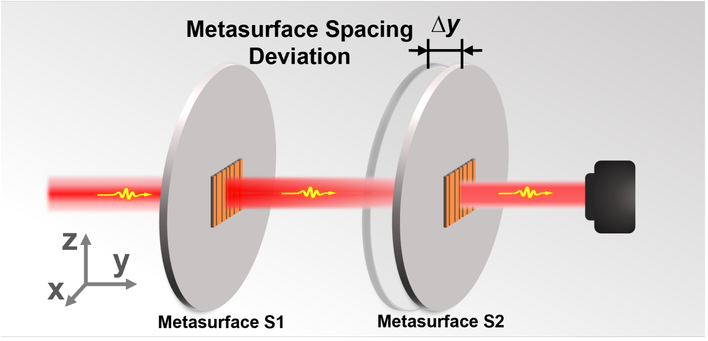

Fig. S19: Misalignment configuration resulting from Metasurface Spacing Deviation.

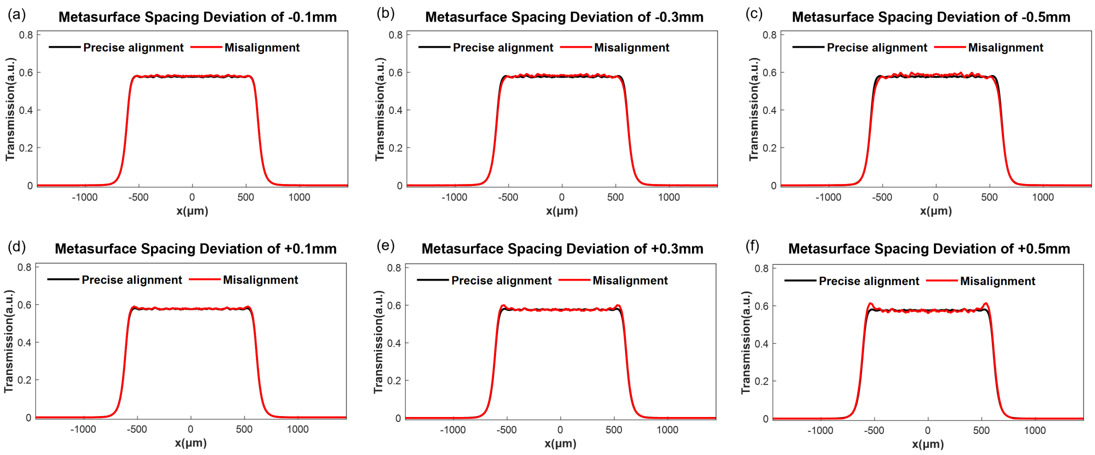

Fig. S20: Flat-top beam irradiance profile with Metasurface Spacing Deviation.

(4) Alignment Deviation

Since the experimental sample involves cylindrical beam shaping, the positional deviation along the z-direction, as shown in the Fig. S21, does not affect the flat-top effect when the light is within the range of the metasurface. Therefore, the positional deviation along the x-direction is the primary source of alignment error. Analysis results indicate that when the positional deviation is within $\pm 18\ \mu m$, the expected beam shaping effect can be achieved, although the uniformity of the flat-top beam's energy profile may slightly deteriorate. This error can be optimized through experimental adjustments.

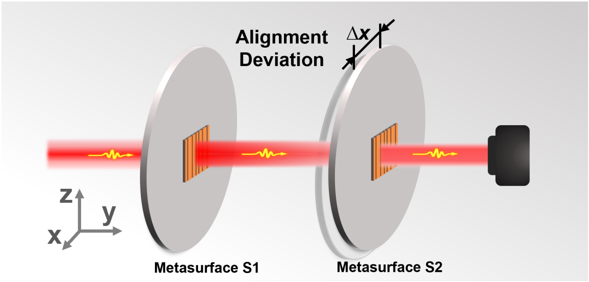

Fig. S21: Misalignment configuration resulting from Alignment Deviation.

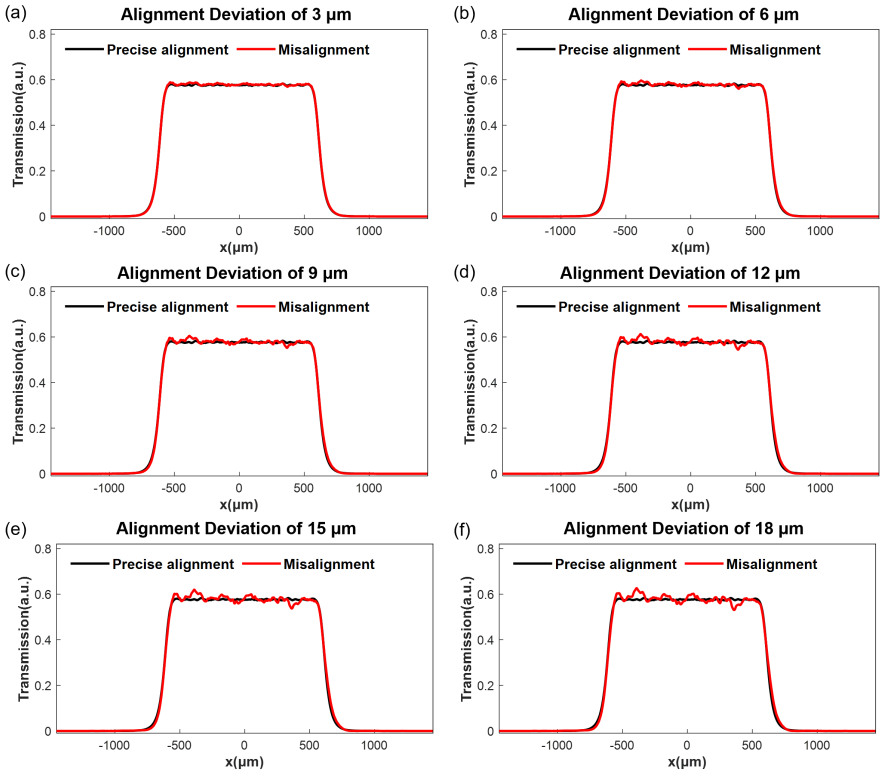

Fig. S22: Flat-top beam irradiance profile with Alignment Deviation.

(5) Yaw Angle Deviation

There may be angular errors during the assembly of the two cascaded metasurfaces, however, yaw angle errors resulting from rotation around the x-axis do not affect the energy profile of the central ray's output. The effect on rays deviating from the center can be equated to a deviation in spacing, which is minimal and does not require focused analysis. When there is a yaw angle error along the z-axis between the two metasurfaces, as shown in the figure, the phase can be understood as a projection relation with the ideal phase. As shown in the analysis, within a ±5° range of angular deviation, the flat-top effect is satisfactory, but there is a shift in the spot position from the ideal location. Therefore, rough alignment of this angle through mechanical means is sufficient to meet the requirements.

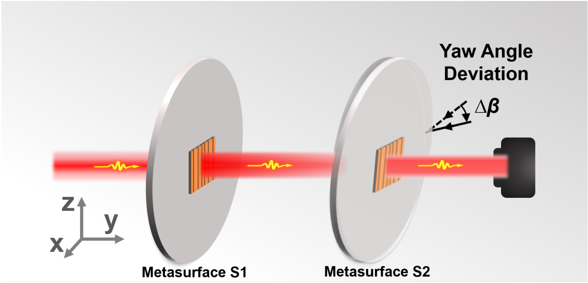

Fig. S23: Misalignment configuration resulting from Yaw Angle Deviation.

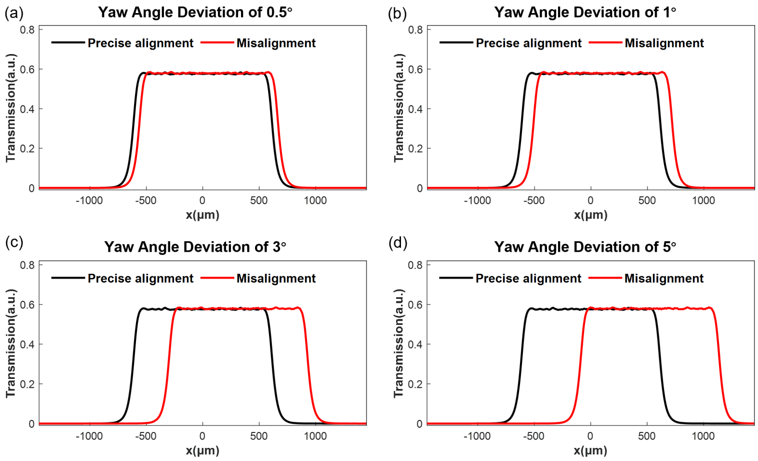

Fig. S24: Flat-top beam irradiance profile with Yaw Angle Deviation.

(6) Rotational Angle Deviation

There may also be rotational angle errors in the two metasurfaces. The positional deviation of light due to the phase modulation by metasurface S2 can be understood as a change in the phase sampling interval compared to the ideal result, which is related to the rotation angle. Analysis results indicate that when the rotational angle deviation

is within ±5°, there is no significant change in the flat-top effect. Therefore, rough mechanical alignment of this angle is sufficient to meet the requirements.

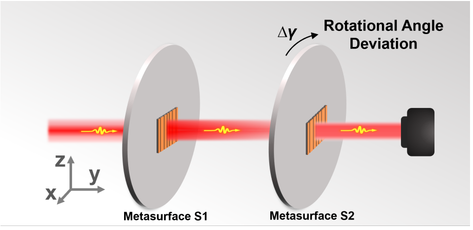

Fig. S25: Misalignment configuration resulting from Rotational Angle Deviation.

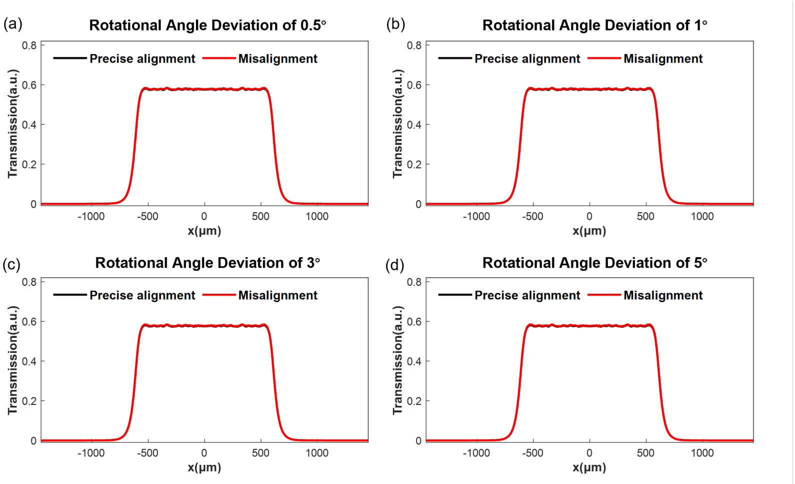

Fig. S26: Flat-top beam irradiance profile with Rotational Angle Deviation.

(7) Detector Position Deviation

When the output beam propagates in free space, diffraction occurs, so the distance of the detector from metasurface S2 also affects the flat-top effect. As shown in the analysis, within a range of ±0.5 mm, there is no significant difference in the flat-top effect, which also validates that the system can produce a collimated flat-top beam. Therefore, the detector can meet the requirements through simple mechanical alignment.

To achieve micrometer-level positioning alignment, the experimental setup in this paper employs three motorized stages (with a minimum increment of 0.05 μm) for aligning the laser with the metasurface, aligning the two metasurfaces, and adjusting their spacing, with the adjustment precision meeting the requirements of the tolerance analysis. The positioning accuracy of the light relative to the metasurface is ensured by concentric mechanical parts, and can be further adjusted by four screws. Commercial optical mounts are used to adjust the yaw and rotation angles of the metasurface. As the experimental test in this paper is for cylindrical metasurfaces, precise longitudinal

alignment is not required. For convenience in adjustment, a manual lifting platform is used for height adjustment.

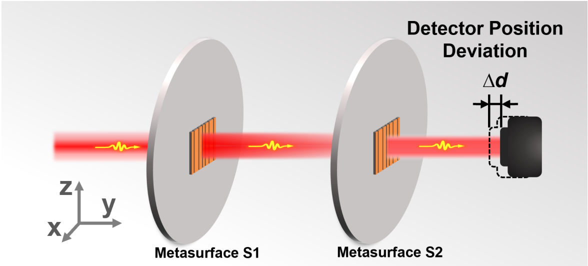

Fig. S27: Misalignment configuration resulting from Detector Position Deviation.

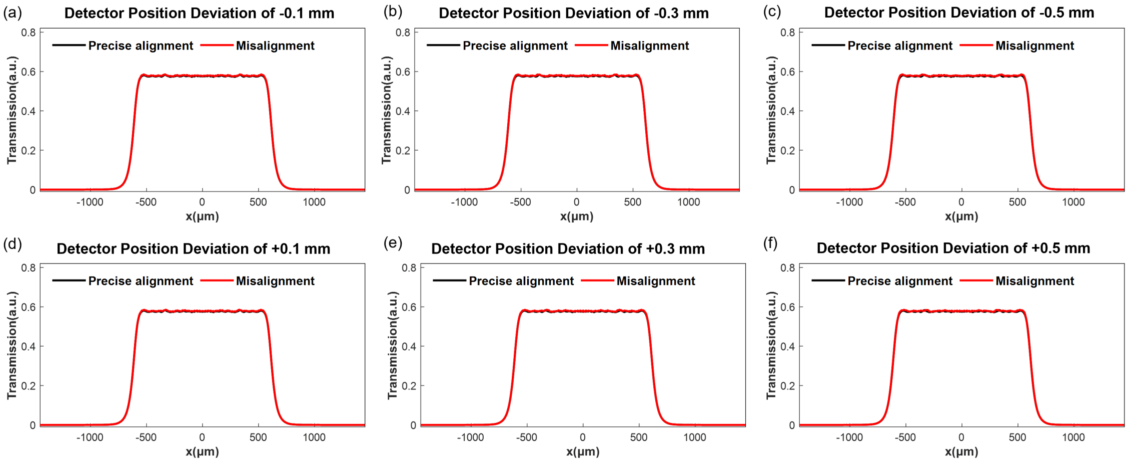

Fig. S28: Flat-top beam irradiance profile with Detector Position Deviation.

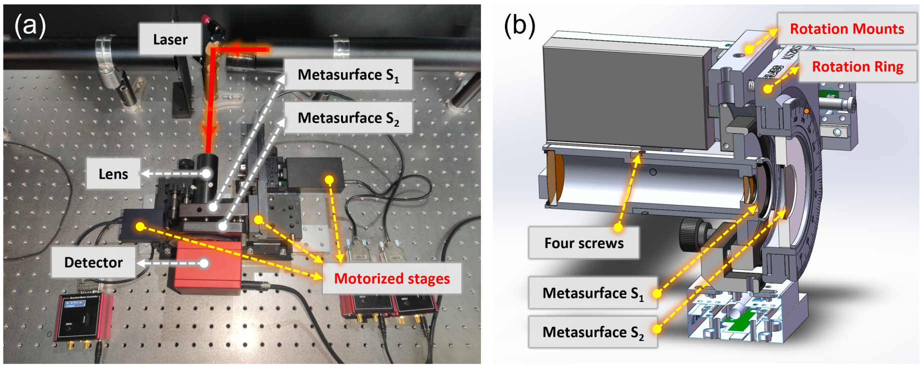

Fig. S29: Alignment tools in experimental characterization systems.

References

[1] L. Hsu, M. Dupré, A. Ndao, J. Yellowhair, and B. Kanté, "Local phase method for designing and optimizing metasurface devices," *Optics Express*, vol. 25, no. 21, pp. 24974-24982, 2017, https://doi.org/10.1364/OE.25.024974

[2] L. J. Allen and M. P. Oxley, "Phase retrieval from series of images obtained by defocus variation," *Optics Communications*, vol. 199, no. 1-4, pp. 65-75, 2001, https://doi.org/10.1016/S0030-4018(01)01556-5
